# Supplementary material for: Mosquito Population Dynamics and Blood Host Associations in Two Types of Urban Greenspaces in Coastal Florida
Source: Insects. 2025 Feb 20;16(3):233. doi: 10.3390/insects16030233 (PMC11942672; doi:10.3390/insects16030233)
Supplement: Supplementary file 1 [file insects-16-00233-s001.zip › Supplementary Data 1.pdf]

**Supplementary Data 1.** Primer sequences and combinations listed in the Reeves et al. 2018 publication. They were used in this study to amplify specific DNA barcoding regions of the *cytochrome c oxidase subunit I* (*COI*) gene during blood meal analysis.

| **Primer label** | **Sequence** | **Specificity** |
|---|---|---|
| Mod_RepCOI_F | 5'- TNT TYT CMA CYA ACC ACA AAG A -3' | Vertebrate universal |
| Mod_RepCOI_R | 5'- TTC DGG RTG NCC RAA RAA TCA -3' | Universal |
| VertCOI_7194_F | 5'- CGM ATR AAY AAY ATR AGC TTC TGA Y -3' | Vertebrate universal |
| VertCOI_7216_R | 5'- CAR AAG CTY ATG TTR TTY ATD CG -3' | Vertebrate universal |

| **Primer combination** | **Amplicon length (bp)** |
|---|---|
| Mod_RepCOI_F + Mod_RepCOI_R | 664 |
| VertCOI_7194_F + Mod_RepCOI_R | 395 |
| Mod_RepCOI_F + VertCOI_7216_R | 244 |
