# Supplementary material for: Mosquito Population Dynamics and Blood Host Associations in Two Types of Urban Greenspaces in Coastal Florida
Source: Insects. 2025 Feb 20;16(3):233. doi: 10.3390/insects16030233 (PMC11942672; doi:10.3390/insects16030233)
Supplement: Supplementary file 1 [file insects-16-00233-s001.zip › Supplementary Data 4.pdf]

Supplementary Data 4. Results from Spearman's rank correlation between the total daily number of mosquitoes collected and cumulative precipitation (inch) accumulated over different periods

Accumulation periods for rainfall prior to trapping (days)	Spearman's Rho	p-value	Significance
7	-0.0570607	0.5575	Not significant
14	0.1132872	0.2431	Not significant
21	0.2692423	0.0048	Significant
