# Supplementary material for: Mosquito Population Dynamics and Blood Host Associations in Two Types of Urban Greenspaces in Coastal Florida
Source: Insects. 2025 Feb 20;16(3):233. doi: 10.3390/insects16030233 (PMC11942672; doi:10.3390/insects16030233)

Supplementary Data 5. Bar graph showing the total number of female mosquitoes collected for all sampling dates at different sites. This is plotted with average daily temperature (F, red line) and daily total precipitation (inch, grey line). Study site names are abbreviated as CP (Charles Park), ML (Moose Lodge), LG (Indian River Lagoon Greenway), and OR (Oslo Riverfront Conservation Area).

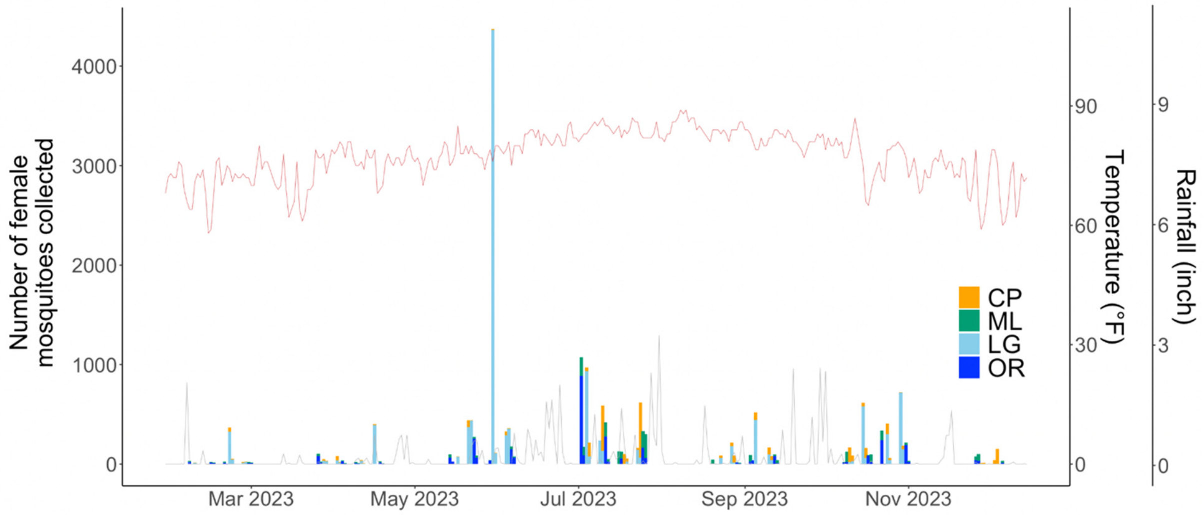

Supplement: Supplementary file 1 [file insects-16-00233-s001.zip › Supplementary Data 5.pdf]
