# Supplementary material for: Mosquito Population Dynamics and Blood Host Associations in Two Types of Urban Greenspaces in Coastal Florida
Source: Insects. 2025 Feb 20;16(3):233. doi: 10.3390/insects16030233 (PMC11942672; doi:10.3390/insects16030233)
Supplement: Supplementary file 1 [file insects-16-00233-s001.zip › Supplementary Data 6.pdf]

Supplementary Data 6. Detailed results of Generalized Linear Model analyses and model diagnostics investigating climatic and habitat factors influencing counts of five mosquito species of interest. All data analyses were performed in R and Rstudio. curainprev21d stands for rainfall accumulated over 21 days prior to trapping (inch), DAvgTemp stands for daily averaged temperature (F). Autoregressive term represents count of mosquitoes in the previous trapping week when data was available.

1. **Model results for *Ae. taeniorhynchus***

Call:
glm.nb(formula = log(Mosquito_number + 1) ~ curainprev21d + DAvgTemp +
  autoregressive_term + habitat, data = clean_data, control = glm.control(maxit = 50),
  init.theta = 11.26825978, link = log)

Parameters	Estimate	Std. Error	Z value	p-value
(Intercept)	0.7726	0.1592	4.851	1.23E-06
curainprev21d	-0.0008	0.0367	-0.022	0.9825
poly(DAvgTemp, 2)1	6.2496	1.5594	4.008	6.13E-05
poly(DAvgTemp, 2)2	-2.9715	1.4662	-2.027	0.0427
autoregressive_term	0.0002	0.0001	2.179	0.0293
habitatresidential	-1.8681	0.2451	-7.621	2.51E-14

Model fit statistic	Value
Null deviance	238.21
Residual deviance	112.35
AIC	286.51
Theta (dispersion parameter)	19.7
Std. Error of Theta	34.4
2 x Log-Likelihood	-272.5
Number of Observations (n)	103
Degrees of Freedom	98

Model diagnostics:

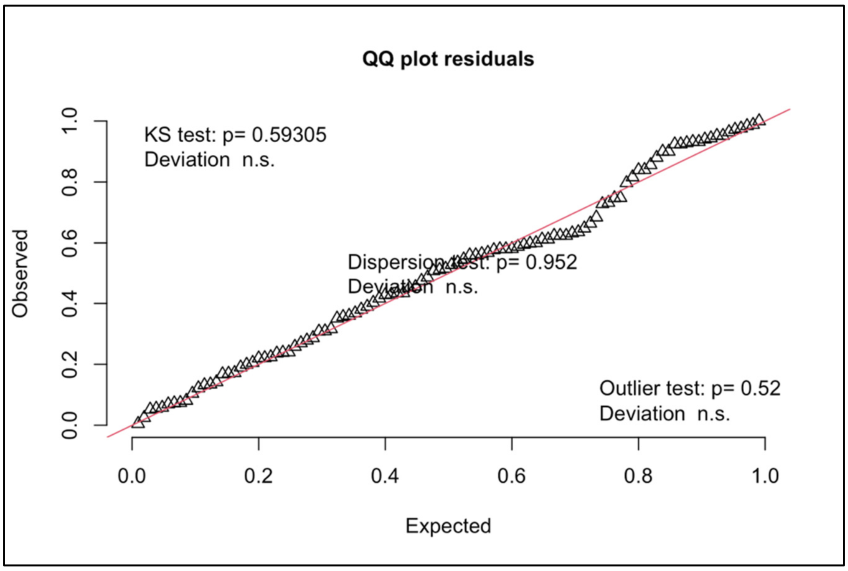

Figure 1: Q-Q plot of model residuals assessing goodness-of-fit. The plot shows the observed versus expected residual distribution with no significant deviations from uniformity (KS test: p = 0.24), dispersion (p = 0.44), or outliers (p = 0.94), indicating an adequate model fit.

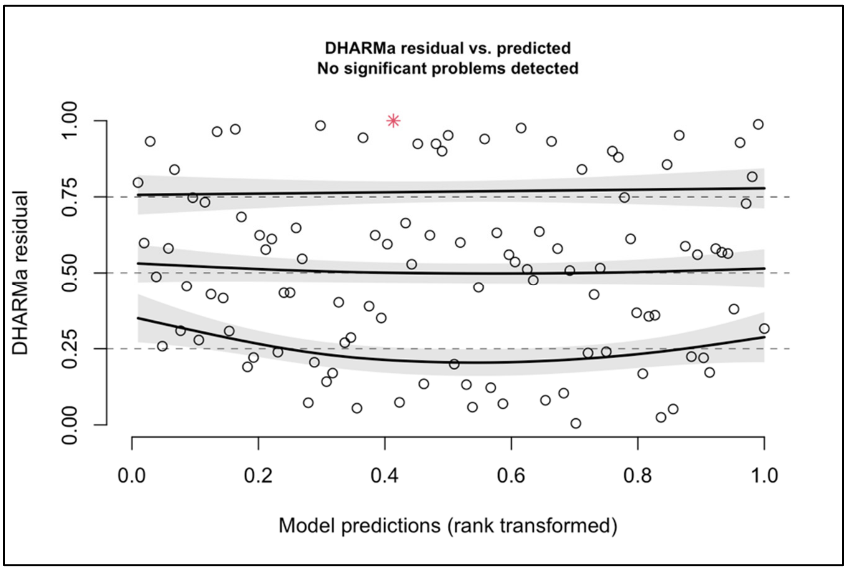

Figure 2: DHARMa residuals versus predicted values for the negative binomial model. The residuals show no significant deviations from the expected distribution, as indicated by the lack of significant quantile deviations. This suggests that the model adequately fits the data without significant issues in residual patterns across the range of predicted values.

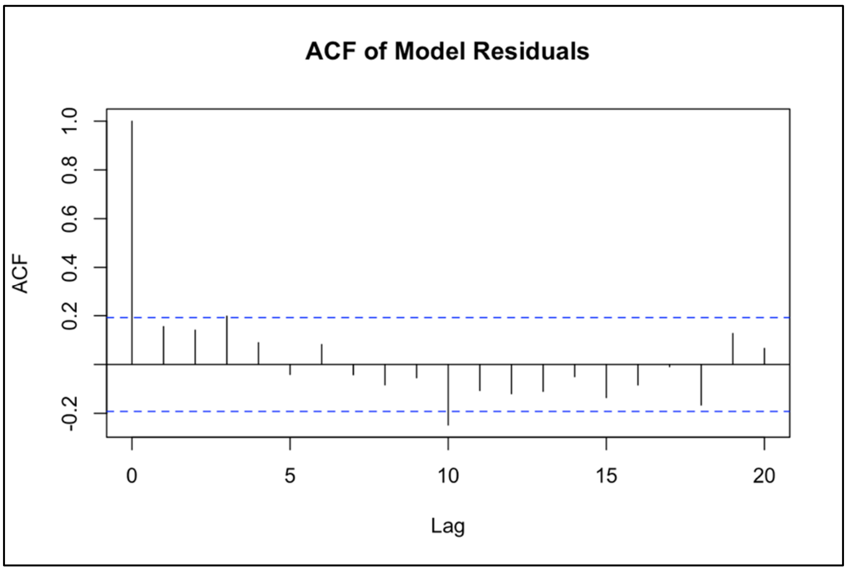

Figure 3 Autocorrelation function (ACF) plot of model residuals. The residuals show no significant autocorrelation at lags beyond 0, as almost all autocorrelation values fall within the 95% confidence bounds (dashed lines). This suggest that the model accounts for temporal dependence in the data, with no significant autocorrelation remaining.

Zero-inflation was not detected in the model, as indicated by the DHARMa zero-inflation test (ratioObsSim = 1.03, p = 0.808). Multicollinearity was assessed using Variance Inflation Factors (VIF), with all predictors showing VIF values well below the common threshold of 5, indicating low multicollinearity.

2. Model results for *Cx. nigripalpus*

Call:
glm.nb(formula = log(Mosquito_number + 1) ~ curainprev21d + DAvgTemp + autoregressive_term + habitat, data = clean_data, control = glm.control(maxit = 50), init.theta = 11.26825978, link = log)

Parameters	Estimate	Std. Error	Z value	p-value
(Intercept)	3.0497	0.2825	10.795	< 2e-16
curainprev21d	0.2929	0.0593	4.932	8.13E-07
poly(DAvgTemp, 2)1	2.5252	1.4726	1.715	0.0864
poly(DAvgTemp, 2)2	-4.8226	1.5928	-3.028	0.00246
autoregressive_term	0.0037	0.0011	3.258	0.00112
habitatresidential	-0.5148	0.2780	-1.852	0.06409

Model fit statistic	Value
Null deviance	162.84
Residual deviance	125.63
AIC	1011.2
Theta (dispersion parameter)	0.5153
Std. Error of Theta	0.0649
2 x Log-Likelihood	-997.2
Number of Observations (n)	103
Degrees of Freedom	98

Model diagnostics:

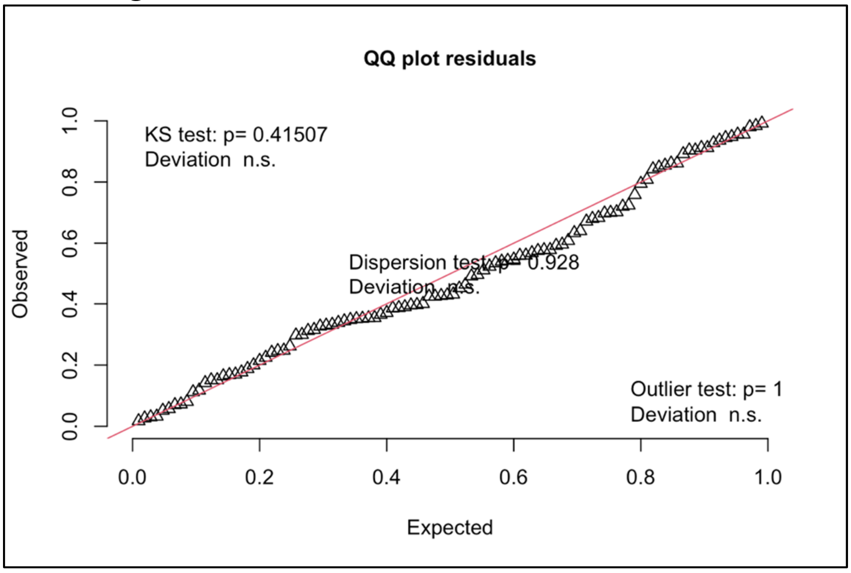

Figure 4: Q-Q plot of model residuals assessing goodness-of-fit. The plot shows the observed versus expected residual distribution with no significant deviations from uniformity (KS test: p = 0.4), dispersion (p = 0.9), or outliers (p = 1), indicating an adequate model fit.

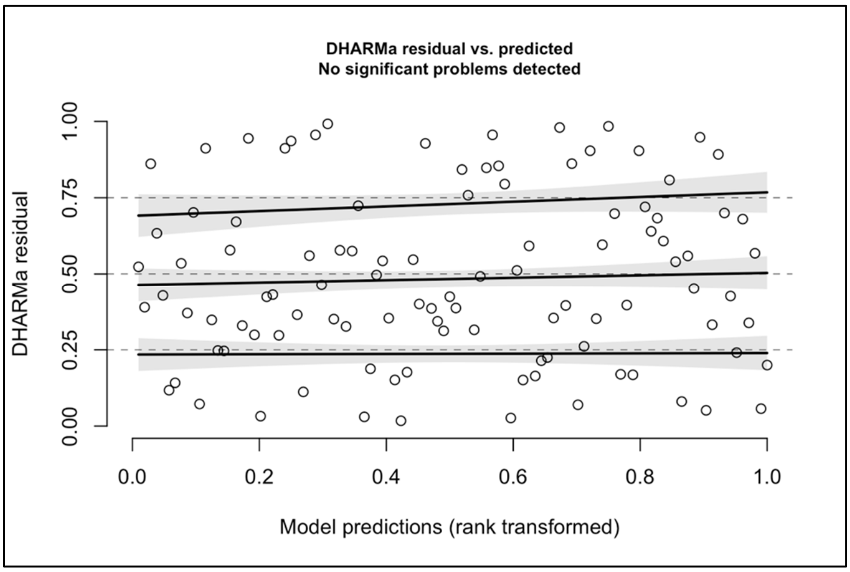

Figure 5: DHARMa residuals versus predicted values for the negative binomial model. The residuals show no significant deviations from the expected distribution, as indicated by the lack of significant quantile deviations. This suggests that the model adequately fits the data without significant issues in residual patterns across the range of predicted values.

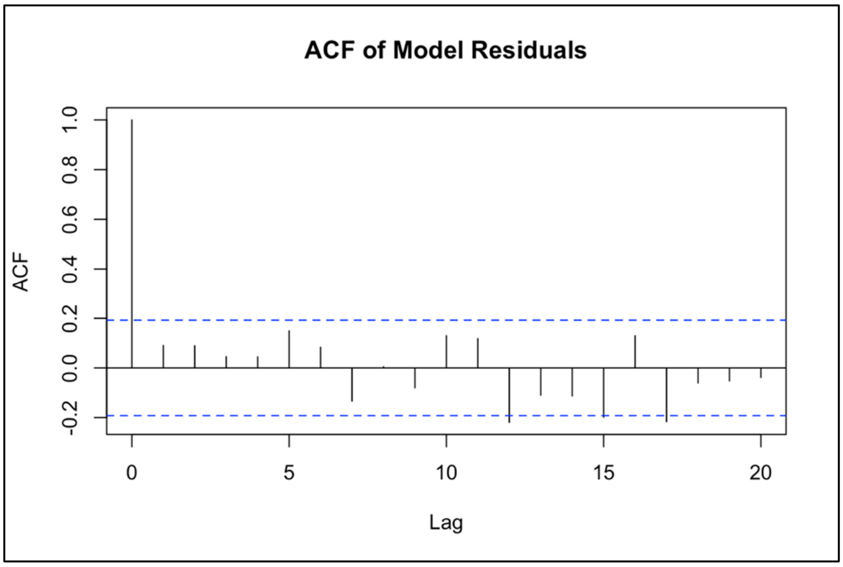

Figure 6 Autocorrelation function (ACF) plot of model residuals. The residuals show no significant autocorrelation at lags beyond 0, as almost all autocorrelation values fall within the 95% confidence bounds (dashed lines). This suggest that the model accounts for temporal dependence in the data, with no significant autocorrelation remaining.

Zero-inflation was not detected in the model, as indicated by the DHARMa zero-inflation test (ratioObsSim = 0.740, p = 0.456). Multicollinearity was assessed using Variance Inflation Factors (VIF), with all predictors showing VIF values well below the common threshold of 5, indicating low multicollinearity.

3. <u>Model results for *Ae. aegypti*</u>

Call:

```
glm.nb(formula = Mosquito_number ~ curainprev21d + DAvgTemp +
  habitat, data = clean_data.g, init.theta = 0.7002155291,
  link = log)
```

Parameters	Estimate	Std. Error	Z value	p-value
(Intercept)	-12.2938	2.7787	-4.424	9.68E-06
curainprev21d	0.0269	0.0706	0.381	0.703047
DAvgTemp	0.1362	0.0352	3.874	0.000107
Habitat-residential	1.9110	0.4092	4.67	3.01E-06

Model fit statistic	Value
Null deviance	117.03
Residual deviance	72.81
AIC	226.77
Theta (dispersion parameter)	0.700
Std. Error of Theta	0.217
2 x Log-Likelihood	-216.8
Number of Observations (n)	103
Degrees of Freedom	100

Model diagnostics:

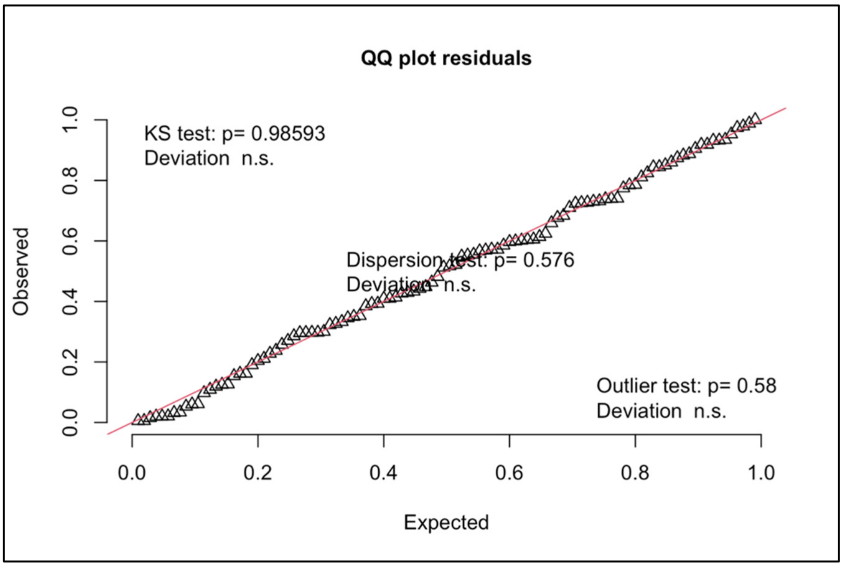

Figure 7: Q-Q plot of model residuals assessing goodness-of-fit. The plot shows the observed versus expected residual distribution with no significant deviations from uniformity (KS test: p = 0.99), dispersion (p = 0.58), or outliers (p = 0.58), indicating an adequate model fit.

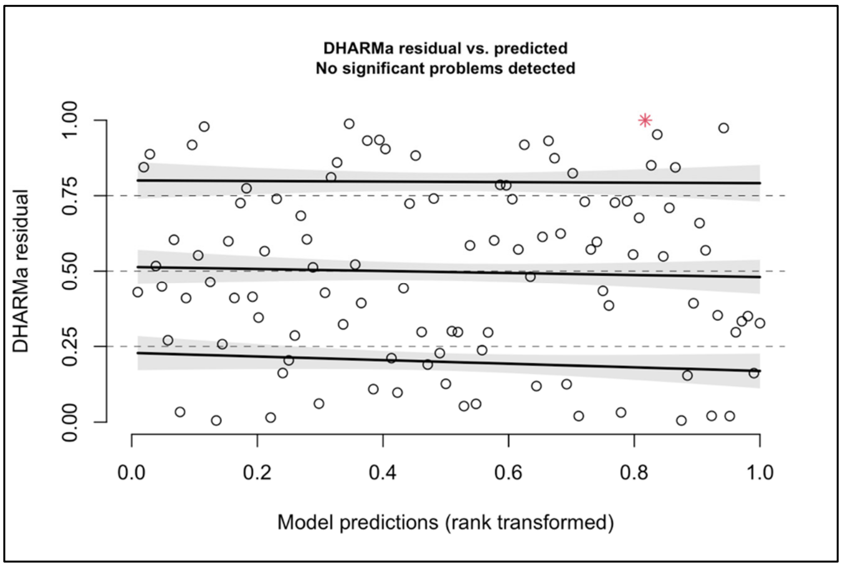

Figure 8: DHARMa residuals versus predicted values for the negative binomial model. The residuals show no significant deviations from the expected distribution, as indicated by the lack of significant quantile deviations. This suggests that the model adequately fits the data without significant issues in residual patterns across the range of predicted values.

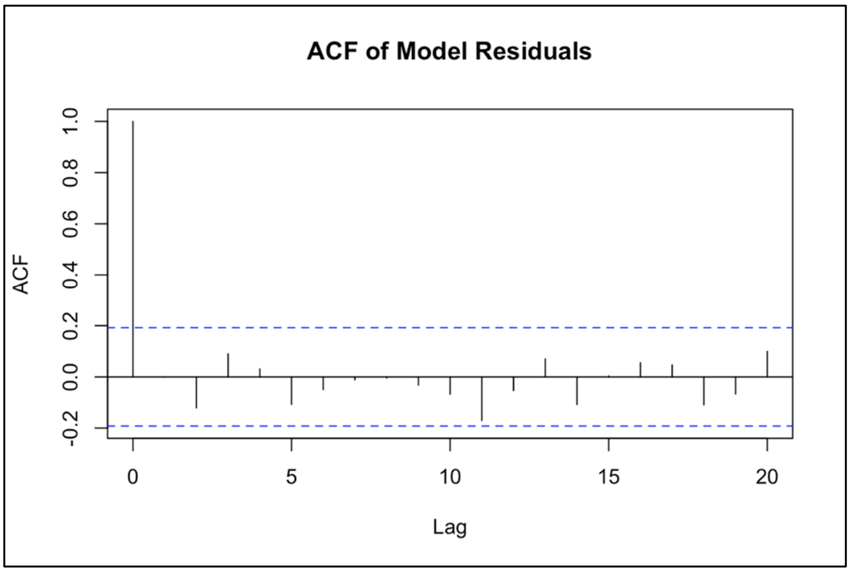

Figure 9 Autocorrelation function (ACF) plot of model residuals. The residuals show no significant autocorrelation at lags beyond 0, as almost all autocorrelation values fall within the 95% confidence bounds (dashed lines). This suggest that the model accounts for temporal dependence in the data, with no significant autocorrelation remaining.

Zero-inflation was not detected in the model, as indicated by the DHARMa zero-inflation test (ratioObsSim = 0.958, p = 0.544). Multicollinearity was assessed using Variance Inflation Factors (VIF), with all predictors showing VIF values well below the common threshold of 5, indicating low multicollinearity.

4. Model results for *Ae. albopictus*

Call:
glm.nb(formula = Mosquito_number ~ curainprev21d + DAvgTemp +
habitat, data = clean_data.g, init.theta = 0.7002155291,
link = log)

Parameters	Estimate	Std. Error	Z value	p-value
(Intercept)	-9.9562	2.9866	-3.334	0.0008
curainprev21d	0.0877	0.0764	1.148	0.2510
DAvgTemp	0.1052	0.0380	2.766	0.0056
habitatresidential	1.0006	0.4278	2.339	0.0193

Model fit statistic	Value
Null deviance	86.39
Residual deviance	67.88
AIC	179.46
Theta (dispersion parameter)	0.628
Std. Error of Theta	0.288
2 x Log-Likelihood	-169.5
Number of Observations (n)	103
Degrees of Freedom	100

Model diagnostics:

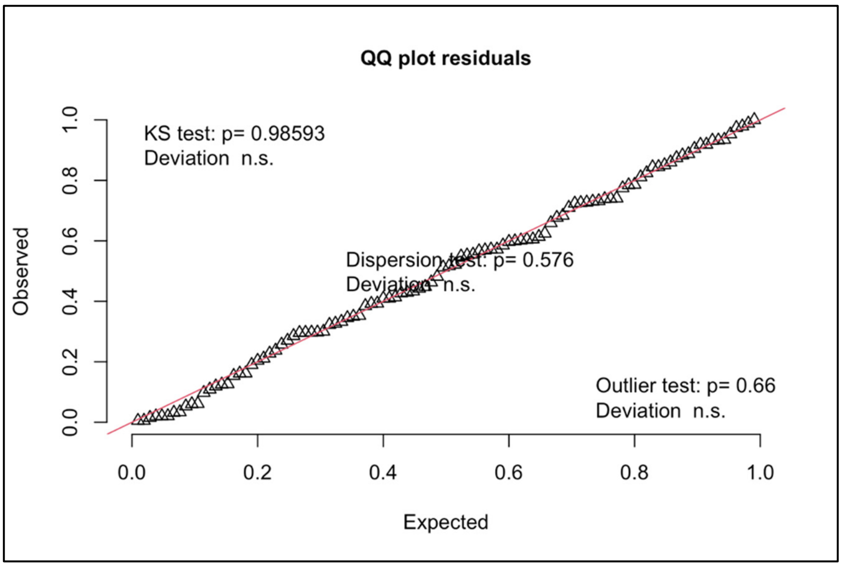

Figure 10: Q-Q plot of model residuals assessing goodness-of-fit. The plot shows the observed versus expected residual distribution with no significant deviations from uniformity (KS test: p = 0.99), dispersion (p = 0.58), or outliers (p = 0.66), indicating an adequate model fit.

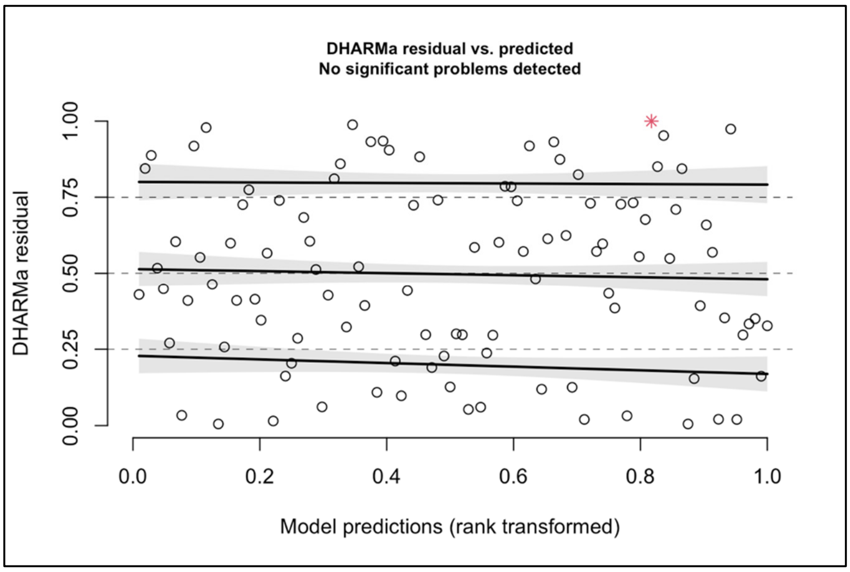

Figure 11: DHARMa residuals versus predicted values for the negative binomial model. The residuals show no significant deviations from the expected distribution, as indicated by the lack of significant quantile deviations. This suggests that the model adequately fits the data without significant issues in residual patterns across the range of predicted values.

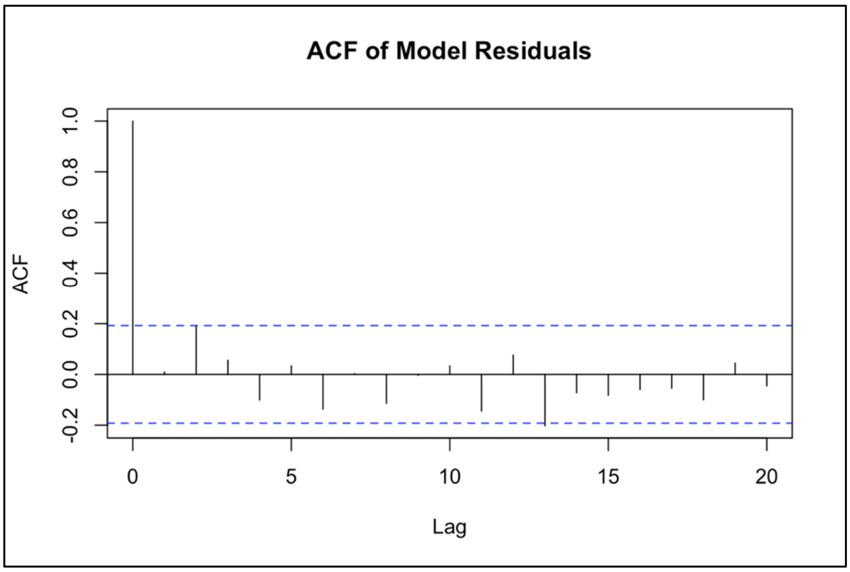

Figure 12 Autocorrelation function (ACF) plot of model residuals. The residuals show no significant autocorrelation at lags beyond 0, as almost all autocorrelation values fall within the 95% confidence bounds (dashed lines). This suggest that the model accounts for temporal dependence in the data, with no significant autocorrelation remaining.

Zero-inflation was not detected in the model, as indicated by the DHARMa zero-inflation test (ratioObsSim = 1.011, p = 1). Multicollinearity was assessed using Variance Inflation Factors (VIF), with all predictors showing VIF values well below the common threshold of 5, indicating low multicollinearity.

pseudo_r2(model.albo)
[1] 0.2143429

5. **<u>Model results for *Cx. quinquefasciatus*</u>**

Call:
glm.nb(formula = Mosquito_number ~ curainprev21d + DAvgTemp +
habitat, data = clean_data.g, init.theta = 0.7002155291,
link = log)

Parameters	Estimate	Std. Error	Z value	p-value
(Intercept)	-2.2039	2.2756	-0.969	0.3328
curainprev21d	0.1898	0.0819	2.316	0.0205
DAvgTemp	0.0228	0.0300	0.76	0.4474
habitatresidential	0.6871	0.4180	1.644	0.1002

Model fit statistic	Value
Null deviance	94.79
Residual deviance	82.53
AIC	337.66
Theta (dispersion parameter)	0.263
Std. Error of Theta	0.059
2 x Log-Likelihood	-327.7
Number of Observations (n)	103
Degrees of Freedom	100

Model diagnostics:

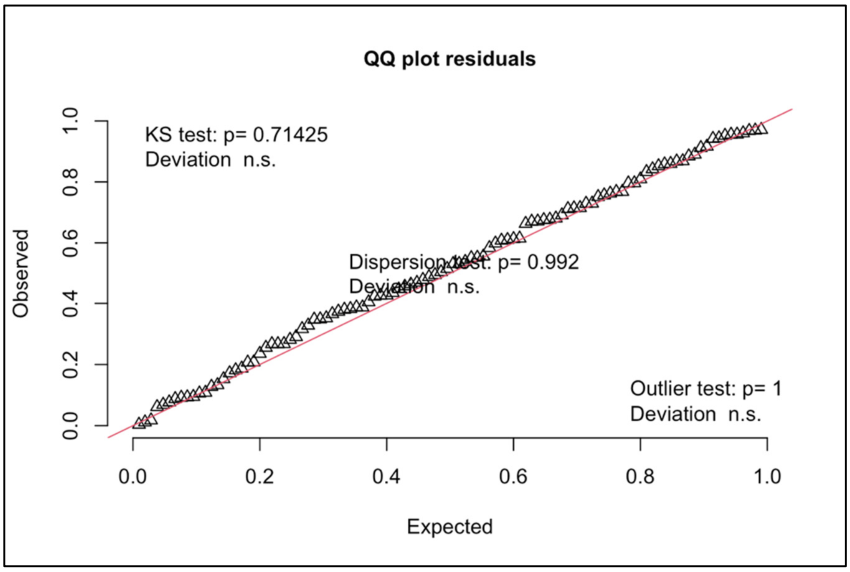

Figure 13: Q-Q plot of model residuals assessing goodness-of-fit. The plot shows the observed versus expected residual distribution with no significant deviations from uniformity (KS test: p = 0.71), dispersion (p = 0.99), or outliers (p = 1), indicating an adequate model fit.

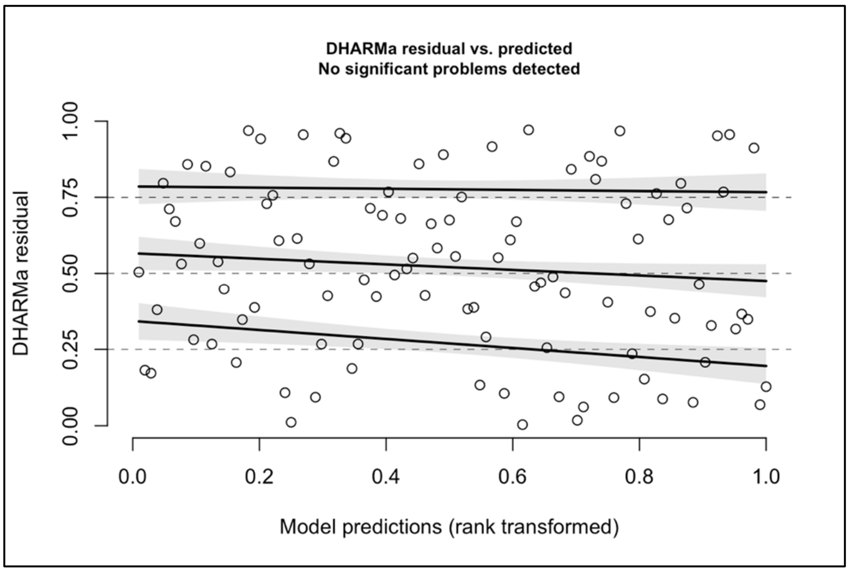

Figure 14: DHARMa residuals versus predicted values for the negative binomial model. The residuals show no significant deviations from the expected distribution, as indicated by the lack of significant quantile deviations. This suggests that the model adequately fits the data without significant issues in residual patterns across the range of predicted values.

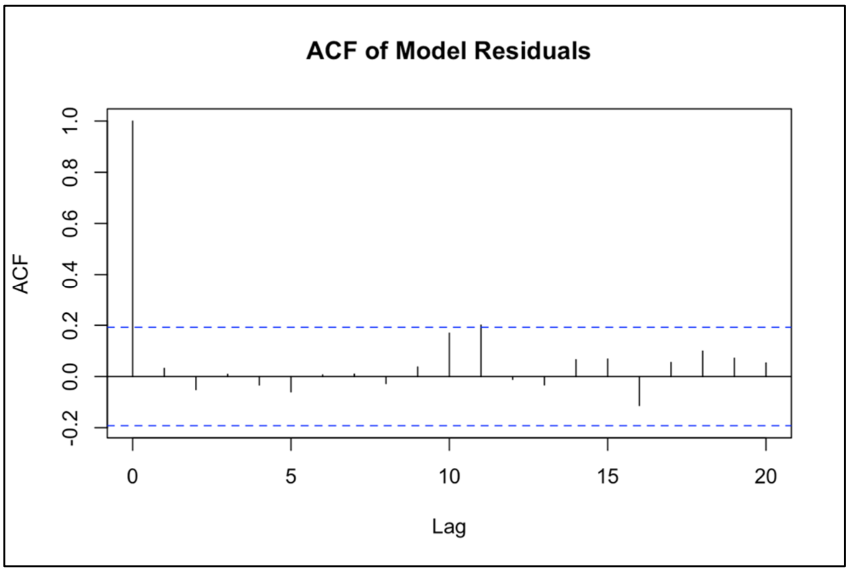

Figure 15 Autocorrelation function (ACF) plot of model residuals. The residuals show no significant autocorrelation at lags beyond 0, as almost all autocorrelation values fall within the 95% confidence bounds (dashed lines). This suggest that the model accounts for temporal dependence in the data, with no significant autocorrelation remaining.

Zero-inflation was not detected in the model, as indicated by the DHARMa zero-inflation test (ratioObsSim = 0.994, p = 1). Multicollinearity was assessed using Variance Inflation Factors (VIF), with all predictors showing VIF values well below the common threshold of 5, indicating low multicollinearity.
