# Supplementary material for: Mosquito Population Dynamics and Blood Host Associations in Two Types of Urban Greenspaces in Coastal Florida
Source: Insects. 2025 Feb 20;16(3):233. doi: 10.3390/insects16030233 (PMC11942672; doi:10.3390/insects16030233)
Supplement: Supplementary file 1 [file insects-16-00233-s001.zip › Supplementary Data 9.pdf]

Supplementary Data 9. Results showing contingency tables and Pearson's Chi-squared test to determine associations between mosquito species and blood meal hosts.

Contingency Table 1. Numbers of *Aedes taeniorhynchus* and all other mosquito species with blood meal from mammals and other hosts

	Mammals	Other Hosts
Ae. taeniorhynchus	20	3
Other mosquito species	27	43

Contingency Table 2. Numbers of *Culex nigripalpus* and all other mosquito species with blood meal from birds and other hosts

	Birds	Other Hosts
Cx. nigripalpus	24	24
Other mosquito species	11	34

Table 3. Pearson's Chi-squared test results (with Yates' correction)

Association between	X-squared	df	p-value
Ae. taeniorhynchus - mammal	14.336	1	0.0002
Cx. nigripalpus - bird	5.4197	1	0.0199
